# Reactivation in Working Memory: An Attractor Network Model of Free Recall

**DOI:** 10.1371/journal.pone.0073776

**Published:** 2013-08-30

**Authors:** Anders Lansner, Petter Marklund, Sverker Sikström, Lars-Göran Nilsson

**Affiliations:** 1 Department of Numerical Analysis and Computer Science, Stockholm University, Stockholm, Sweden; 2 School of Computer Science and Communication, Department of Computational Biology, KTH (Royal Institute of Technology), Stockholm, Sweden; 3 Stockholm Brain Institute, Stockholm, Sweden; 4 Department of Psychology, Stockholm University, Stockholm, Sweden; 5 Department of Psychology, Lund University, Lund, Sweden; Heidelberg University, Germany

## Abstract

The dynamic nature of human working memory, the general-purpose system for processing continuous input, while keeping no longer externally available information active in the background, is well captured in immediate free recall of supraspan word-lists. Free recall tasks produce several benchmark memory phenomena, like the U-shaped serial position curve, reflecting enhanced memory for early and late list items. To account for empirical data, including primacy and recency as well as contiguity effects, we propose here a neurobiologically based neural network model that unifies short- and long-term forms of memory and challenges both the standard view of working memory as persistent activity and dual-store accounts of free recall. Rapidly expressed and volatile synaptic plasticity, modulated intrinsic excitability, and spike-frequency adaptation are suggested as key cellular mechanisms underlying working memory encoding, reactivation and recall. Recent findings on the synaptic and molecular mechanisms behind early LTP and on spiking activity during delayed-match-to-sample tasks support this view.

## Introduction

Working memory (WM) is a general-purpose system for concurrent processing and short-term maintenance (STM) of no longer externally available information in the service of higher-order cognition [1]. Current neural network models of WM have typically focused on the STM component in the context of “subspan” memory load suggesting that information is retained by persistent neural activity in a recurrent network with connectivity formed and intrinsic cell excitability modulated by previous experience [2], [3].

However, direct measures of persistent activity in delayed-match-to-sample tasks in monkeys show that neuronal firing rate increases occurring during working memory (e.g. during the delay period of working memory tasks) are typically modest, or may show trends in firing rate (i.e. firing rates which increase or decrease during the course of the delay) [4]. Individual cells show substantial variability in their firing behaviour across trials, and firing frequency also varies markedly over the course of a single trial. Furthermore, multiple items cannot easily be activated simultaneously in a recurrent neural network with lateral inhibition, because this will tend to produce a rivalry situation resulting in convergence to one of the competing representations, typically corresponding to the closest attractor state [5]. Moreover, the persistent activity type of model may not be sufficient for characterizing human WM processes operating during complex behaviour, often requiring simultaneous encoding and integration of new input while maintaining a larger set of internal representations in accessible form.

Free recall of word-lists is a classic experimental paradigm requiring flexible coordination of encoding, rehearsal and reactivation during word-list learning and subsequent recall [1], [6]. A large amount of shared variance between immediate free recall and complex WM span task performance has been reported, which implies common mechanisms [7], the nature and plausibility of which can be explored by neural network models. By using “supraspan” list-lengths that exceed the narrow capacity-limits of the phonological STM buffer a number of ubiquitous phenomena emerge, including the U-shaped serial position curve denoting enhanced memory for items from the beginning (primacy effect) and the end (recency effect) of a study list relative to items from the middle of the list [8]. Within traditional dual-store models, primacy arises because the first few items are sufficiently rehearsed via STM to be transferred to episodic long-term memory (LTM) stores. Recency is interpreted as reflecting “unloading” of the last few items which are assumed to still be rehearsed in the STM buffer when recall begins, making them directly accessible for output [9]. The mid-list items are most susceptible to forgetting because they cannot be rehearsed enough to enter LTM and will be displaced from the STM buffer before recall which causes the asymptote in the serial position curve. The order in which items are recalled constitutes another important behavioural phenomenon. In particular, subjects are more likely to successively recall items that were presented nearby each other during list learning (temporal contiguity effect), and to recall such neighbouring list items in the same order as they were encoded rather than in the reversed order, e.g. [10], [11]. These phenomena provide additional behavioural constraints to models of list learning, e.g. [11].

We propose here an abstract neurocomputational recurrent attractor memory model to account for human experimental data on immediate free recall. This model provides a plausible resolution of the above mentioned problems with persistent activity models of STM, by viewing WM as encoded by fast and volatile Hebbian synaptic plasticity and modulated non-Hebbian intrinsic excitability. The changed connectivity in combination with the adaptation properties of the network units results in a dynamically changing activity in the form of spontaneous rapid hopping between the patterns in WM. No consensus has yet been reached regarding the precise mechanisms underlying free recall dynamics. In particular, to the best of our knowledge none of the previously proposed models have used rapid reactivation of stored patterns as the key mechanism to understand free recall dynamics. A number of different accounts have been proposed.

Models accounting for free recall can broadly be classified into models that are predominantly constrained by behavioural or by biological data. The majority of free recall models published so far, falls into the behaviourally constrained models that primarily aim to characterize “mental algorithms” capable of reproducing human-like recall dynamics quantitatively while disregarding biophysical details on how they are neurally implemented in the brain. These models, sometimes also referred to as abstract computational models have been postulated to explain key empirical data, e.g. by invoking dual-stores [12], or a time-varying context representation (e.g. [13]–[15]). Biologically constrained models are typically based on neural networks, where the behavioural phenomena can be induced from the dynamics of the network, for example retrieval of memories in free recall data. Only a few attempts of biologically constrained modelling of the primacy and recency effects exist. Burgess, Shapiro and Moore [16] used a Hopfield attractor network to model serial recall, where primacy was accounted for by reinforcement (multiplying) of previously stored weights, whereas recency was modelled by weights boundaries. Wong, Kahn et al. [17] used a diluted asymmetric Hopfield network to account for primacy and recency. However, their model could only account for the primacy effect given that the initial distribution of weights were different from the asymptotic distribution following learning of a large number of patterns, indicating very particular constraint for the phenomenon to be modelled. Green, Prepscius, and Levy [18] suggested a simplified biologically motivated neural model. However, their model accounted either for primacy, or recency but not both, suggesting two distinct different systems. Recency was accounted for without rehearsal. Sandberg et al. [19] proposed and studied a neural network model of WM based on fast and volatile Hebbian learning demonstrating recency without addressing the phenomenon of primacy. Sikström [20] accounted for primacy, recency, and the isolation effect (enhanced learning of odd items) with a single mechanism related to a biologically plausible learning rule. This form of learning suggests an adaptive threshold for induction of LTP and LTD, where primacy is accounted for by an increased LTP following a low threshold in the beginning of the list. Despite the relative lack of models of working memory based on synaptic plasticity and reactivation dynamics, some models of serial recall share similar synaptic and neuronal properties and produce similar activity-jumping dynamics between sequence elements (e.g. [21]).

Here we present a biologically constrained attractor memory network model of free recall based on the model of Sandberg et al. [19], [22], which was extended with a slow synaptic trace variable (z) as described below. We demonstrate how its function and dynamics are generated from biological mechanisms of fast and volatile Hebbian plasticity and modulated non-Hebbian intrinsic excitability, together with neuronal adaptation. Our simulation results demonstrate that this kind of attractor memory model can remarkably well reproduce key data on immediate free recall of word-lists. The model provides detailed mechanisms and a unifying principle behind storage, distributed reactivations and recall during single-trial word-list learning.

## Methods

### An abstract memory network model

We implemented the above outlined mechanisms in a modular recurrent neural network model of a Potts type architecture [22], [23]. The network configuration used has H modules of M units and is assumed to represent a small piece of cortex. Model units correspond to local cortical neuronal populations comprising on the order of a hundred neurons, e.g. functional minicolumns and the larger modules are generalized hypercolumns or macrocolumns, i.e. bundles of minicolumns interacting mainly via lateral inhibition, such that each hypercolumn module acts as a soft winner-take-all microcircuit [24], [25]. The biological rationale behind this model is further discussed below, after the presentation of the model itself.

Our non-spiking units or “minicolumns” have graded output in [0 1] and feature a slow adaptation with a time constant of 2.7 seconds, matching approximately data on the relaxation time constant of the calcium transient underlying the hippocampal sAHP in humans [26]. The units within a hypercolumn interact via divisive normalization of activity, i.e. a form of lateral inhibition. The Bayesian-Hebbian learning rule (BCPNN) used was derived from Bayes rule with the rationale that a postsynaptic unit computes an estimate of its posterior likelihood of being active given the activity of its presynaptic units weighted by the respective connection strengths [22], [27]. This learning rule has previously been used in the context of efficient scalable associative memory [28] and as a palimpsest type of synaptic short-term memory [19]. Notably, the learning rule is of a “three-factor” type, i.e. it contains a “print-now” parameter that regulates the plasticity (learning rate) of the network, akin to the action of e.g. dopamine D1 receptor activation [29], [30].

The units in the network are recurrently connected to themselves, to mimic the local recurrent excitation within a functional minicolumn, and, more importantly, also to all other units in the network. Such a dense long-range connectivity is not quite biologically plausible but is necessary for the stable operation of a network of the limited size used here. Typically, a set of sparse activity patterns are stored in the network by means of strengthened excitatory connections within such a pattern and inhibitory connections between patterns. The resulting positive feedback within the memory patterns forms attractors of the memory dynamics and the divisive activity normalization within the hypercolumns prevents runaway excitation. The active units are subject to a slow build-up of adaptation which terminates activity after some hundreds of milliseconds.

The levels of activation (*s*), adaptation (*a*) and output (*o*) of each model unit are updated according to the following equations (see Tables 1 and 2 for values of parameters and initial values of state variables):
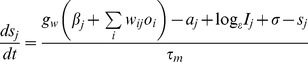
(1)

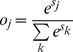
(2)

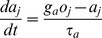
(3)Here *g_w_* is the gain of connections, *g_a_* is the adaptation gain, *τ_m_* is a unit time constant, *τ_a_* is the adaptation time constant, *I_j_* is the input to unit *j*, and *σ* represents the amplitude of zero mean Gaussian noise added to the support of each unit in every iteration. Eqn. 1 represents the time evolution of the activation of units due to synaptic input, adaptation, external input and noise. Eqn. 2 describes the normalization of activity performed within each hypercolumn. This represents the lateral inhibition provided by local basket cells. Eqn. 3 describes the typically slow time evolution of the unit adaptation.

**Table 1:** Model variables.

Variable	Description	Initial value
*a_j_*	Adaptation of unit *j*	0
*s_j_*	Support of unit *j*	log(1/M)
*o_j_*	Activity of unit *j*	1/M
*I_j_*	Input	[*ε* 1]
*z_i_*	Source unit activity trace	1/M
*z_j_*	Target unit activity trace	1/M
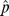	Global variable trace	0
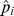	Source unit activity trace of connection from unit *i* to *j*	1/M
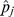	Target unit activity trace of connection from unit *i* to *j*	1/M
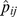	Co-activity trace of connection from unit *i* to *j*	1/M^2^
*β_j_*	Bias of connections to unit *j*	*g_w_*log (1/M)
*w_ij_*	Weight of connection from unit *i* to *j*	0

**Table 2:** Model parameters.

Parameter	Description	Default value
*H*	N:o hypercolumns	12
*M*	N:o units per hypercolumn	12
*Δt*	Simulation time step	0.001 s
*τ_m_*	Unit time constant	0.050 s
*τ_a_*	Adaptation time constant	2.70 s
*σ*	Zero mean Poisson noise	100 Hz/0.20
*g_a_*	Adaptation gain	97.0
*g_w_*	Weight gain	2.00 (encoding), 1.70 (recall)
*g_b_*	Bias gain	12.0
*τ_zi_*	Presynaptic trace time constant	0.240 s
*τ_zj_*	Postsynaptic trace time constant	0.240 s
*τ_p_*	Weight update time constant	10.0 s
*κ*	Print-now parameter	1.10 (encoding) 0.00 (recall)
*Θ*	Recall threshold	11.0
*ε*	Numeric single precision limit	1.17549 10^−38^

The learning rule is based on occurrence and co-occurrence statistics collected during encoding as running averages with different time constants. The pre- and post-synaptic trace variables (*z*) represent presynaptic and postsynaptic events involved in synaptic plasticity like e.g. NMDA channel opening (*z_i_*) and membrane depolarization due to back-propagating action potentials (*z_j_*). These traces serve as a short-term memory buffer allowing modification of connections between units that are activated with some time lag. This tends to bind together activations that occur consecutively. The z-traces typically have relatively short time constants (*τ_zi_*, *τ_zj_*) and are updated according to the following equations:
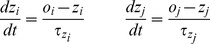
(4)


The tuning of the z-trace time constants was an important step towards reproducing the memory recall data.

The estimated probabilities of pre- (*i*) and post-synaptic (*j*) activation and co-activation (*p*) are updated as:
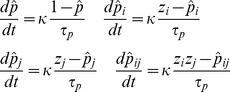
(5)


The connections weights (*w*) and unit biases (*β*) are continuously computed based on these *p*-estimates according to the Bayesian-Hebbian learning rule (Sandberg, Lansner *et al.*, 2002) as:
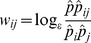
(6)

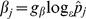
(7)where



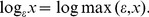
(8)There is a separate longer time constant for the weights and bias dynamics (*τ_p_*), a print-now parameter that gates learning (*κ*), as well as a gain for the unit bias (*g_β_*). The parameter *ε* represents a low cut off value to avoid log of 0.

It needs to be pointed out that our learning rule produces excitatory connections between units with correlated activation as well as inhibitory connections between units with anti-correlated activity. This change in polarity obviously violates known neurobiology, but is remedied by assuming that the inhibition is disynaptic, mediated via a local inhibitory interneuron close to the target pyramidal cell, rather than via a direct pyramidal-pyramidal synapse.

The model was implemented in C++, parallelised using MPI (Message Passing Interface), and run on an IBM Blue Gene/L cluster with 1024 dual core nodes. This allowed us to simulate 1024 list experiments at the same time with an execution time of less than 5 minutes. The model was integrated using the forward Euler method with an integration step size of 1 millisecond. It was carefully checked that results were not sensitive to a doubling of the time step to 2 milliseconds.

### Neurobiological rationale for the model

The above described highly abstract computational model can in several respects still be related to contemporary data and theories about cortical functional architecture [31]–[33]. It has repeatedly been suggested that the cortex comprises a mosaic of modules, such as functional columns or other types of sub-networks [24], [25], [34]–[36]. Such local networks would have a diameter of a few tens of microns and their excitatory neurons would be more densely connected among themselves than to the surrounding ones and would likely be selectively targeted by afferent fibers from thalamus. They may be spatially segregated as cortical minicolumns or may be anatomically diffuse (i.e., intermingled with other similar modules) [37]. A modular organization at this level is not prominent in rodents but more so in e.g. cats and primates where minicolumns are more anatomically distinct [38]. Such a partitioning in functional sub-networks has also been suggested to generate a patchy long-range cortical connectivity [39], [40].

Other data indicate that these local functional sub-networks are organized in bundles to form maps or larger modules with a diameter on the order of a few hundreds of microns (i.e. macrocolumns, hypercolumns, or barrels) [41]. Fast spiking local inhibitory interneurons (e.g. basket cells) provide inhibitory feedback within such a module. It has been suggested that this introduces competition and operation somewhat like a local soft winner-take-all module. A hypercolumn may be assumed to represent, in a discretely coded fashion, some attribute of the external world. For instance, in the primary visual cortex the orientation or direction of an edge stimulus at a certain position on the retina is represented by elevated activity in a corresponding orientation column. This also leads to sparse activity in the network, on the order of about 1–5%. Such an activity level is in accordance with overall activity densities which may be even below 1% in higher order cortex [42]. The learning rule used in our abstract model generates both positive and negative weights between minicolumn units (eqn. 6). In the real cortex, excitatory connectivity would be supported by direct pyramidal-pyramidal synapses, whereas long-range inhibition would likely be disynaptic, e.g. from a pyramidal cell onto a local vertically projecting and dendritically targeting interneuron, e.g. a double bouquet cell.

A prominent feature of real pyramidal cells which is also represented in this model is their adaptation, which can be spike frequency adaptation or other e.g. calcium dependent forms. In fact, also synaptic depression which is not represented in our model, produces a similar phenomenon in spiking cortex models [31]. Both of these processes tend to terminate ongoing activity, like for instance reactivations, in a recurrent network. A further feature specific to our abstract model and absent in most other abstract models is the bias term of the learning rule. In a theoretical sense this term corresponds to the *a priori* probability of activation given previous experience, so that a unit which has been often active in the past will have an elevated spontaneous base level of activation already without input. This type of process has been described in real neurons in terms of “intrinsic excitability” and is one among several neurobiological mechanisms of activity dependent regulation [2], [43].

### Methods summary of behavioural data

The present work is based on data from the Betula Study [44], [45], a prospective cohort study on memory and health. A total of six independent, randomly selected samples with a total of more than 4500 persons are included in the Betula study. Recruitment were conducted by a random sample of the population, for details see Nilsson et al. [44], [45]. As the basis for the present study, we selected one sample of the Betula Study, consisting of 500 subjects in the age range of 35 to 55 years with an average of 45 years. These data are from Sample 1 that was tested for the first time in 1988–1990. Participants diagnosed as demented were excluded by following a well-established procedure.

The Betula study consists of a large battery of cognitive tests [45]; however, the data in the present study are taken from one task involving study and immediate free recall of a word-list. Participants studied a list of 12 unrelated nouns with the instruction of a free recall test after the final word of the list. The words were presented auditorily at a rate of one item per 2 sec and the participants were instructed to recall orally as many words as possible in any order they preferred during a period of 45 sec, in keeping with classical studies of free recall (e.g., Murdock [46]). There were four parallel lists available and participants were counterbalanced across these four lists. Word frequency for each list was 98 words per million words (range 50–200). There were four different conditions with respect to the attentional demands in this task. A card-sorting task was given as a distracter (1) both at study and test, (2) at study only, (3) at test only, or (4) neither at study nor at test. The data used here were from this final condition with focused attention at both study and test.

For further information on the Betula study, see Appendix S1.

### Modelling free recall data

The single-trial word-list learning task, with list-length 12, was modelled consistent with the experimental setup as follows. Each word was represented by a sparse random pattern with one unit active in each hypercolumn. A new input was presented to the network every two seconds. It was fed to the network by clamping its units to the input for one second (*κ* = 1.1) where after input gain and *κ* were set to zero for one second until the next list item was presented.

**Figure 1:**
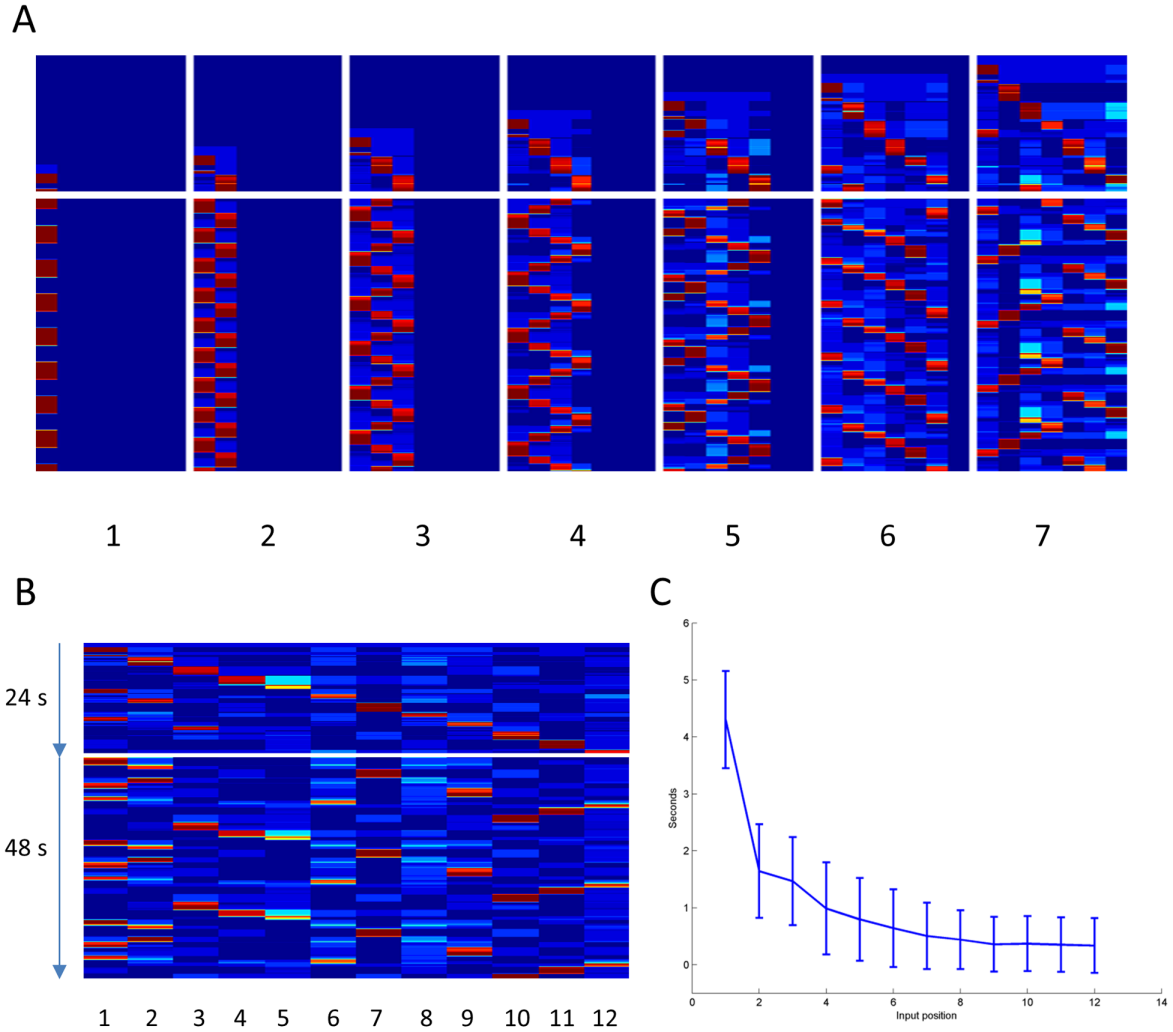
Item activation and reactivation. A: Activity during storage and recall for different number of stored patterns (1–7 indicated below each display). Blue-red color shows activity (m-values) with dark blue representing 0 and deep red 1. Time is on the vertical axis, item number on the horizontal. The horizontal spacing separates activity during encoding (above spacing) and spontaneous recall (below spacing) in each display. B: m-values during the presentation and recall of a 12 item list. Each item is presented during 2 seconds, in total 24 seconds, followed by a 48 seconds spontaneous recall period. C: The average reactivation time and its standard deviation during presentation (y-axis) as a function of item position (x-axis).

The list presentation thus lasted for 24 seconds. After the last item was presented 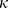 was kept at zero for 45 seconds while the network spontaneously reactivated (recalled) items from memory (Figure 1A). The network was fully reset before the next list was presented.

Recall of an item was detected by continuously calculating the normalized dot product between the reactivated pattern and each stored pattern *k* (eqn 9).
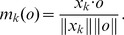
(9)Here the vector *x_k_* is one of the list patterns and the vector *o* is the activity of the network. A time series of *m_k_* values was obtained as this equation was applied to the network activity at every time step. The *m_k_* values were accumulated in each time step and if the k:th accumulated sum passed a threshold (*θ* Table 2) a successful recall of item *k* was registered. This procedure defined the time and order of recall of items.

Typically the same item was recalled repeatedly but only the first recall was registered. Subsequent recalls still happened in the network and these recalls could act to suppress other first recalls. Statistics were collected for traditional serial position effects and the serial output order over items to assess contiguity effects or conditional response probability (CRP); i.e., the probability that neighbouring list items during encoding are recalled together (see below). Since the time step was 1 millisecond the recall of two items at exactly the same time was highly unlikely. If occurring, this list was excluded from the statistics.

The conditional response probability (CRP) was calculated as the fraction of times that a recalled item was followed by recall with a certain lag, i.e. difference of the input position of the currently recalled item and the subsequently recalled item [47]. Positive values represent forward recall transitions (e.g. a lag of “+1” denotes recalls of two list items in the same order as they were presented at encoding) and negative values backward recall transitions (i.e. input order is reversed at output).

For each set of parameters tested, 1024 list simulations were performed and the output data analyzed in terms of recall length distribution, primacy-recency curve, and CRP curve. The set of parameters that had the minimal mean square error between experimental and simulated data was selected. A large number of simulations were run to find a good fit.

## Results

### Storing single and multiple memories

As a first demonstration of the operation of the network model as a short-term working memory we ran a number of simulations where one up to seven word patterns were stored in the network. Initially, the stimuli were presented once in succession, where after a recall period of 48 seconds was allowed (Figure 1A). The figure shows the overlap (*m_k_*) over time between network activity and each of the word patterns. As can be seen, the neural activity during the recall period is highly dynamic. This can be understood from the interplay of the synaptic connectivity developed during the encoding phase and the adaptation properties of the neural network units. When a stimulated word pattern activates a set of units while keeping others silent, the simultaneously active units become connected by excitatory (Hebbian) synapses whereas lateral inhibition increases between pairs of units where one is active and the other silent (eqn, 5,6). The recurrent excitation within memorized word patterns promotes their activation and the strong lateral inhibition between them prevents co-activation resulting in competition between the patterns. Active units adapt, i.e. get slowly hyperpolarized due to e.g. spike frequency adaptation, and after a couple of hundred milliseconds they can no longer sustain activity and the pattern inactivates. This disinhibits the rest of the network thus allowing some other fresh memorized pattern to activate. The ensuing competition process, determining exactly which memory gets activated next, produces a winner within few tens of milliseconds, where after the same excitation-adaptation cycle is repeated. In all cases shown in Figure 1A, all encoded memories were recalled multiple times.

In the case of a single stored item, there was sometimes persistent activity but most often a slow oscillation over the entire recall period. As soon as more than one item was stored recall took the form of repeated reactivation of the memories. This spontaneous jumping of activity results from the above described interplay between recurrent excitation, adaptation and activity normalization. The dwell time of an activated memory and its total active time decreased, and the time interval between reactivation of a memory items increased with memory load, i.e. the number of items stored in WM.

Already during the encoding phase, previously encoded items tended to reactivate between subsequent item presentations. This reactivation strengthened the synapses in the reactivated pattern thus enforcing its encoding and increasing the probability of activation during the free recall phase, i.e. promoting primacy.

In the case illustrated here, reactivation would continue forever since there were no further new items presented. However, as this model is in essence a palimpsest type of memory, newly presented items tend to overwrite already stored ones resulting in their forgetting [22]. The capacity of this short-term memory, i.e. the number of items kept as the reactivated set, can be regulated by changing the time constant of connections (*τ_p_*), which in this case was 10 seconds.

### Encoding and recall dynamics in the model

Figure 1B shows the dynamics resulting from presenting a list of 12 word items as described above. As in the previous case, in between later stimulated items there was also spontaneous reactivation of list items presented earlier in the list. The activity during the recall phase illustrates how early and late items dominate recall over those in the middle of the list. When this type of simulation was repeated many times with new word patterns we found that the average time of reactivation during the word presentation phase decreased from early to late presented words (Figure 1C). However, there was considerable trial-to-trial variability due to noise imposed as well as the different overlap structure in pattern sets in different trials.

**Figure 2:**
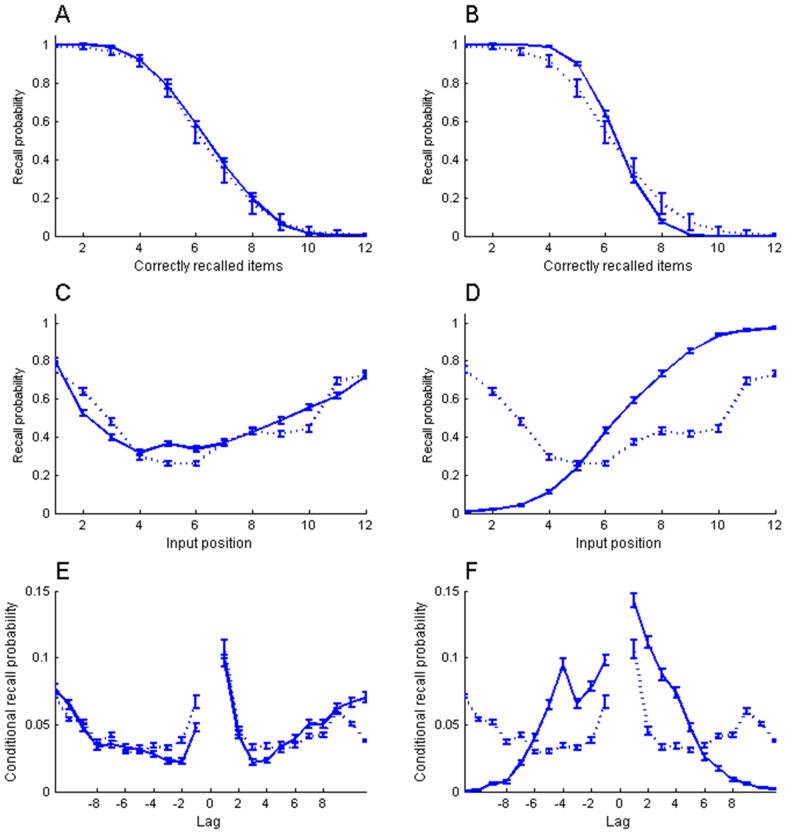
Recall statistics. Comparison of model recall results with and without reinstatement during encoding (left and right column panels respectively). Dotted curves show experimental data from the Betula study. A, B: Memory span function (number of items on the x-axis and cumulative recall on the y-axis). C, D: Primacy-recency curve, i.e. probability of recall versus input position (recall probability on the y-axis). E, F: Conditional recall probability (CRP) curves. Probability on the y-axis and lag, i.e. difference in item input order, on the x-axis.

We then collected statistics over 1024 simulated list-learning experiments. Figure 2A compares the experimental and simulated recall performance, i.e. the probability of correctly recalling the number of items given on the x-axis from the list, disregarding order of recall. Figure 2B shows the same function with blocked reactivation during encoding in the model, which is intended to simulate divided attention. The comparison to the same experimental data as in 2A demonstrates that the effect of this manipulation is only minor. Figure 2C shows that the first and last presented words had a higher probability of recall than words in the middle of the lists, i.e. the network model displayed clear primacy as well as recency effects. The correspondence with experimental data from human subjects is quite good. The kind of recency effect seen here has been demonstrated earlier in several other modelling studies including Sandberg et al. (2003), where it was due to the same mechanism, i.e. the memory traces of the last few items in the list are spared from retroactive interference from following list items.

The primacy effect is not as straightforward to understand as recency. Our current explanation is that it occurred because of the temporally distributed reactivation of previously presented items, preferably occurring in the early part of the list presentation where there is less competition from rehearsal of other items. A typical example can be seen in Figure 1B where the first item is reactivated twice and the second and third list items once each during the list presentation. The reactivation of an item is typically not immediate since the presentation of the item itself induces some adaptation (see Methods eqn 3) which needs to decay in order to allow reactivation.

This key role of item reactivation is supported by the fact that blocking this reactivation during the presentation period removed entirely the primacy effect (Figure 2D). Notably, the primacy and recency effects were quite similar to what is seen in averaged data from a large sample of human subjects.

Words presented next to each other at encoding were more likely to be recalled together with a bias towards forward asymmetry in recall transitions (Figure 2E), which is consistent with previouis empirical findings, e.g. by Kahana [47]. This so called contiguity effect was also facilitated by reactivation during encoding, however, blocking the reactivation diminished, but did not eliminate this effect (Figure 2F). The mechanism behind this contiguity effect is that there are remaining synaptic traces from a previous list item when the next item in the list is stimulated and the print-now signal elevated. This will create a weak but significant weight increase that later manifests itself as an elevated probability of recall of neighboring list items. Reacitivation may faciliate this effect.

**Figure 3:**
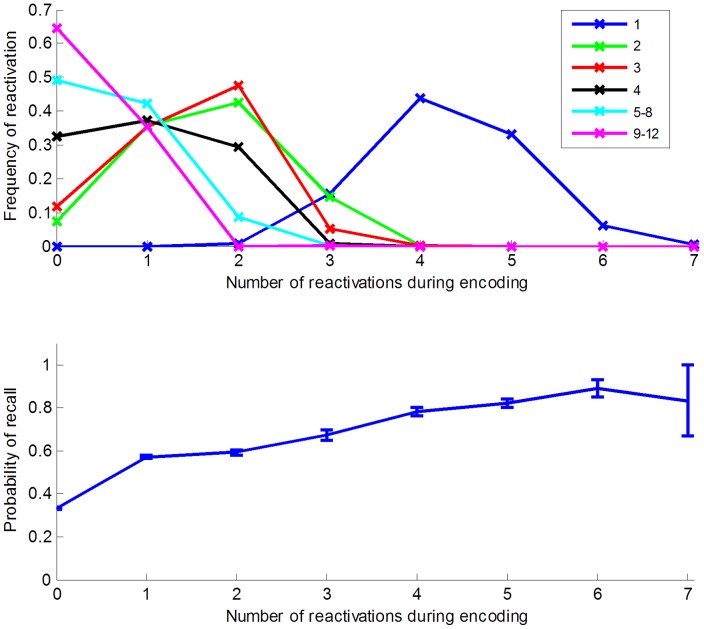
Reactivation determines recall probability. Upper panels: Distribution of reactivation over item input position. Items 1–4 are shown individually, items 5–8 and 9–12 are grouped. Lower panel: Probability of recall of an item as a function of number of reactivations during encoding.

In order to better understand the relation between item reactivation and primacy we further studied in more detail how the number of reactivations of an item depended on its input position. This showed clearly that reactivations occurred predominantly for the early items (Figure 3 upper). It was quite pronounced for the first item and gradually became less prominent, thought list items at input positions two and three were still reactivated significantly more than later list items. Furthermore, the probability of recall of an item increased with the number of reactivations during the preceding encoding period (Figure 3 lower). This result fits well with empirical data from overt rehearsal free-recall paradigms, in which participants are instructed to continuously report aloud which items they rehearse. Such experiments have demonstrated that item rehearsals tend to be temporally distributed throughout the ensuing list presentation [6]. This suggests that primacy depends more on the frequency, distribution and recency of item reactivations during the study phase [48], [49] than on continual rehearsal time. Because early list items have more overall time, and fewer competitors with respect to accessing WM during initial processing, they are eligible to become reactivated more often and in a more distributed fashion than later items.

## Discussion and Conclusions

We have proposed a new kind of real-time neural network model of WM, where storage is based on fast expressing and volatile Hebbian synaptic plasticity and modulated intrinsic excitability. Current data on fast forms of plasticity of synaptic strength as well as intrinsic excitability modulation suggest that such a mechanism of WM is indeed a possibility [2], [43], [50].

This relatively simple model replicates the experimental data from human word-list learning remarkably well. It suggests a mechanistic explanation at the level of neuronal and synaptic processes of a fundamental cognitive function and also challenges previous neuronal network models of WM solely based on persistent activity. This hypothesis of synaptic WM is compatible with previous results of e.g. visual delayed-match-to-sample since when only one item is stored, activity during the recall period (comparable to the delay period) is exclusive to that item for many seconds (Figure 1A, leftmost). As more items are stored their internal representations alternates in dominating activity. It is thus mainly when storing multiple items that a WM mechanism based on a synaptic trace and one based on persistent activity predicts different dynamics. The mechanism of hopping between several transient attractor states has been described earlier for non-spiking as well as spiking network models [19], [31], [51] and was designated as “latching transitions” by Russo and Treves et al. (see e.g. [52]–[54]), although these were used in a different context. This proposed alternation between memory patterns being reactivated during inter-stimulus intervals (ISIs) is compatible with recent data in monkeys [55]. In humans, findings from a MEG study by Fuentemilla et al. [56] suggested that oscillatory activity in the theta band was phase-locked to cyclic “replay” of individual items during STM, which tentatively correspond to how alternating reactivations in ISIs would manifest during list learning. In contrast to standard persistent activity WM models, the reactivation dynamics of our proposed synaptic WM model is consistent with the observed results of increasing as well as decreasing unit activities during a memory delay period as well as the relatively modest activity increases seen.

There are certainly also some differences between model and experimental data. One reason might be that we model the average of a population of subjects with just one set of parameters, which in general is not possible. Using separate parameter sets for each subject is obviously not possible due to lack of data. A more elaborate model might assume that subjects belong to sub-groups each one to be represented with a different set of parameters. This is indeed a much more complex approach and was not judged feasible at this stage. Another factor contributing to a limited fit to the experimental data is that the lists in the experiment comprised a relatively limited set of words that were reused between subjects, whereas the simulation model randomly renewed word patterns on every trial. Also this factor is difficult to compensate for in the current setup.

It needs also to be stated that the parameter set reported here was the best found, but there were several others that came quite close in fitting the data. Though no extensive analysis of the parameter space was possible, we observed that quite different parameter combinations could give a similar degree of fit to data. Further, the sensitivity to parameter variations was moderate since results of different runs were similar despite considerable variations of parameters. Our conclusion is that the model is to some degree under-determined by this amount and precision of the data. Future studies should attempt to pinpoint specific aspects of this kind of model and subject each of them to detailed experimental analysis. The model itself could be made more elaborate, e.g. by including interactions with intermediate-term and semantic LTM as well as mechanisms to account for serial order phenomena, weakly represented in the current model. It could also be made more biologically detailed by using a population of spiking units instead of a single graded output unit to represent a cortical minicolumn. Such an extended model is likely to display additional features characteristic of human memory recall, like oscillatory activity in theta, alpha and gamma bands as measured in M/EEG [57], including dependencies on memory load [58]. It is further likely to substitute the crisp on-off activity, as displayed by the current non-spiking model, with low-rate irregular spiking activity of a much more realistic nature, as has been demonstrated in previous modelling work [32].

Our model was tested on some of the most basic findings in single-trial word-list learning; however, we acknowledge that there are a number of other well-studied empirical phenomena that the model has not yet been applied to. For example, the introduction of continuous distracters during list learning is of theoretical importance because it provides a possibility of attenuating rehearsal. Previous findings have shown that this diminishes the primacy effect but maintains the contiguity effects [10]. To investigate this issue we compared two conditions in the Betula data where subject had undivided or divided attention to the word-list encoding task. This showed that the primacy effect was diminished in the divided attention task compared to the undivided task, whereas the CRP curve was less affected by this manipulation. These results are consistent with the result from Howard and Kahana [10], showing that a continuous distracter intervening between each presented list item attenuates the primacy effect but maintains the contiguity effect.

We compared these experimental results with our model, where divided attention was simulated by blocking reactivation during encoding (Figure 2 B, D, F) and we found a clear similarity between them. In the simulation the contiguity effect in the CRP curves is largely dependent on the overlap between synaptic traces from the current and the previous presented item, and is therefore less influenced by blocking of reactivation. In contrast, the primacy effect is largely dependent on reactivation. Thus, the model predicts, consistent with experimental data from the Betula study and Howard and Kahana's results [10], that the primacy effect can be diminished while maintaining a high contiguity effect. Future work will study in detail how our model accounts for these findings, as well as how it responds to other important variables such as immediate/delayed recall, age effects, word semantic and frequency, etc.

Some further aspects of the experiment were poorly represented in the model. For instance, the network is always completely reset between lists. In experiments, subjects were memorizing four lists in sequence, which likely resulted in some proactive interference phenomena between lists that are not captured in our model setup. To study this phenomenon would represent an interesting further extension of this work. We also ignore the multiple reactivations of items in our analysis due to the fact that such information was not available from experimental data. These additional recalls likely affect the recall sequence by suppressing other first recalls. The details of this dynamics would also warrant further study and could give valuable insights into the underlying neural mechanisms.

There are a number of computational models aiming to account for a rich number of behavioural aspects of immediate free recall (e.g., TODAM2, [59], TCM [60], SIMPLE [14]). However, several of them are not neural network models but of a more phenomenological nature. For example, two recent computational models; the SIMPLE (Scale Invariant Memory, Perception, and LEarning) model [14] and The Temporal Context Model (TCM) [60], have proposed temporal distinctiveness and context retrieval, respectively, as “unitary” mechanisms that can account for a range of free recall data without assuming a division between STM and LTM. The SIMPLE model has recently received much theoretical interest in the context of free recall tasks (e.g. [61], [62]). It posits that list items are encoded and represented at different points in a “psychological space” where the time elapsed between input and recall, together with an item's temporal distance(s) from neighbouring list items, is assumed to determine the degree of item discriminability and thus recall probabilities for different serial positions. From the time recall begins (and over subsequent recalls) the temporal distances between earlier input positions are logarithmically compressed as items move into the past whereby their discriminability diminishes over time. Recency is accounted for by relative increases in temporal distinctiveness for later list items and fewer interfering nearby list items. Primacy is attributed to increased discriminability of earlier relative to mid-list items due to fewer neighbouring items [14]. This model does not account for contiguity effects.

The Temporal Context Model (TCM) [60], [63] represents a recent framework extending on earlier context-based theories of free recall (e.g. [64]) suggesting that list learning depends on associating items to an internal context representation that changes gradually over time. Contiguity effects, whereby items encoded at nearby list positions also tend to be recalled together, are accounted for by a shared temporal context established during list learning that is reinstated as retrieval cue during later recall, rather than temporal vicinity per se. The recency effect is explained by the close similarity between the context representations at the end of list learning and the beginning of recall, while the primacy effect is accounted for by a parameter that increases the cue strength of the first item. In contrast, our model does not require a context to simulate the behavioural phenomena (although we agree with the importance of contextual cueing in episodic memory retrieval), and pre/post synaptic traces of presented items are essential to account for contiguity effects. In our view, a neural network type of model with real-time dynamics such as the one studied here provides additional possibilities to include biological constraints, which narrows the number of possible models to further investigate.

At the cognitive level, the proposed model resonates well with a number of psychological models, e.g., Cowan's embedded processes model [65] and Oberauer's concentric 3-layer memory model [66], stating that WM reflects the control of access to a capacity-limited focus of attention (FOA) encompassing maximally active LTM representations and a ‘surrounding’ larger portion of facilitated LTM representations (e.g., recently presented items that have been removed from the FOA but still remain activated above baseline). Converging behavioural evidence suggests that only one element can reside in the FOA at any given moment [67], which agrees with our model, in which only a single item (memory reactivation or new input) can be active at a time. Items outside this FOA are assumed to undergo rapid trace decay and will be lost unless they regain access to the FOA where their activation is strengthened, as occurs during ISI repetitions. Since both reactivation of items and encoding of new input would compete for processing in the one-item FOA, a continuous shifting between externally and internally driven (re)activation of LTM patterns will occur throughout list learning. Interestingly, an fMRI study by Peacock-Lewis et al. [68] moreover found that delay-related sustained activity only represents the item currently held within the FOA rather than all WM contents, and that shifting attention away from the encoded target item (towards information retrieved from LTM or new stimuli) instantly disrupted this activity without any detrimental effects on WM performance. This indicates that items removed from the FOA can be rapidly and efficiently reactivated on demand and that, at least in humans, subspan WM is unlikely dependent on sustained activation.

Finally, our model agrees with psychological theories of free recall proposing that repetitions/reactivations of early list items during subsequent ISIs rely on the same retrieval process as recall [49].

Notably, the synaptic plasticity dependent mechanism of WM put forward here conforms qualitatively to established knowledge about LTP and LTD [69] except with regard to temporal aspects. It thus unifies mechanisms behind short- and long-term forms of memory in a biologically plausible manner that elegantly eschews the need for dual-stores or context based mechanisms to explain many core phenomena in single-trial supraspan word-list learning. This makes it quite straight forward to understand interactions between WM and LTM as well as memory consolidation based on repeated reactivation.

The persistent activity view has dominated neuronal network modelling of WM for quite some time. Earlier and also more recent models have proposed that active maintenance may instead be dependent on some form of fast and volatile synaptic plasticity [19], [58], [70], [71]. However, Mongillo et al.'s [70] and Lundqvist et al.'s [58] models are based on a non-Hebbian mechanism (synaptic augmentation) and are therefore unable to store novel items, whereas the model presented here is based on Hebbian plasticity and is capable of doing this. It is the first dynamic real-time neural network model shown to reproduce the statistics of human immediate free recall of word-lists and it poses a challenge for other mechanistic models of short-term WM to replicate the same kind of data equally well or better.

## Supporting Information

Appendix S1 **The Betula Study.**
(RTF)
