# Supplementary material for: Reactivation in Working Memory: An Attractor Network Model of Free Recall
Source: PLoS One. 2013 Aug 30;8(8):e73776. doi: 10.1371/journal.pone.0073776 (PMC3758294; doi:10.1371/journal.pone.0073776)
Supplement: Appendix S1 — The Betula Study. (RTF) [file pone.0073776.s001.rtf]

APPENDIX S1: The Betula Study
The data on which the present study is based emanate from a prospective cohort study on memory and health - the Betula Study (L-G Nilsson, Adolfsson et al., 2004; L.-G. Nilsson, Bäckman et al., 1997). The Betula Study had its first wave of data collection in 1988. Four waves have since then been completed, and a fifth wave is now in progress for 2008-2010. A total of six independent, randomly selected samples with a total of more than 4500 persons are included in the Betula study. The major aims of the study are (a) to examine the development of memory and health in adulthood and old age, (b) to determine early signs and risk factors for dementia, (c) to assess premorbid memory functioning, and (d) to explore characteristic features of successful aging.
Participants
One sample of the Betula Study (S1) served as the basis of participant selection for the present investigation. The recruitment procedure has been described in detail elsewhere (e.g., Nilsson et al. 2004, 1997 (L-G Nilsson, Adolfsson et al., 2004; L.-G. Nilsson, Bäckman et al., 1997)); only a brief summary will be given here. For each sample, the recruitment of participants started by randomly, and independently for each sample, obtaining names from the population registry in Umeå, a city of about 110 000 inhabitants in northern Sweden. Persons sampled were first contacted by mail and followed up by a telephone call to make appointments for a health examination and memory testing one week later. In this letter, it was explained how the name of each participant had been obtained, that participation was voluntary and could be interrupted at any time, that the health examination (including blood sampling) and the memory testing would each take 1.5 to 2 hours, and that all results would be stored in a database in a coded, unidentifiable format.
Up to the time of T4 (2003-2005) 210 of the participants had been diagnosed as demented. These participants were excluded from the sample used in the present study. Dementia diagnosis followed a well established procedure. 
The participants of Betula have been compared with the population of Sweden with regard to demographic factors (L.-G. Nilsson, Bäckman et al., 1997). Aside from a slightly higher level of education among the participants, due to Umeå being dominated by its university, the study population reflects the general levels of Sweden.
Memory tests
The cognitive tests in Betula were extensively described in Nilsson et al. 1997 (L.-G. Nilsson, Bäckman et al., 1997). Great effort was made to compose the battery of memory tasks in such a way that they were theoretically motivated, and that it would be possible to explore a wide variety of processes and hypothetical memory systems analytically.
The data used in the present study emanate from a task involving study and test of a word listword-list. Participants studied a list of 12 unrelated nouns with the instruction of a free recall test after the final word of the list. The words were presented auditorily at a rate of 2 sec per item and the participants were instructed to recall orally as many words as possible in any order they preferred during a period of 45 sec. There were four parallel lists available and participants were counterbalanced across these four lists. Word frequency for each list was 98 words per million words (range 50-200). There were four different conditions with respect to the attentional demands in this task. A card-sorting task was given as a distracter (1) both at study and test, (2) at study only, (3) at test only, or (4) neither at study nor at test. The data used here were from this final condition with focused attention at both study and test.
Health examination
Each participant was administered (a) an extensive health examination including blood sample testing, (b) an interview about health status and a revised version of Katz ADL, (c) a questionnaire about social and economic issues, and (d) a questionnaire about critical life events.
Determination of dementia in the Betula Project
In the Betula project, determination of dementia diagnosis followed a procedure such that participants that fulfil one or several of the following criteria are referred to a spe- cialist in neuropsychiatry: (a) suspected dementia signs observed by the staff conducting the Betula testing, (b) MMSE performance below 24, or (c) a decline of 3 or more points on the MMSE from the previous testing occasion. The psychiatrist evaluated the participants' dementia status based on the Diagnostic and Statistical Manual of Mental Disorders, Revised Fourth Edition  (DSM_IV-R, 1994). Dementia could be established independently of the Betula testing. However, the medical records of all participants were reviewed to classify participants as either demented or non-demented in the present study, ensuring that no diagnosed dementias were overlooked. The relation between MMSE performance and dementia evaluation enabled us to control for dementia diagnoses prospectively within the 5-year time frame of the study. Differentiating dementia sub-types is intrinsically difficult without bio- logical markers. For this reason, participants were only classified as being either demented or non-demented in the present study. However, according to the present dementia assessment, 74% of the demented participants were eval- uated as having AD or ''AD and/or vascular dementia''. This indicates that AD was the most prevalent dementia category in this sample.
Selection
For the present study we selected from the Betula database data from a total of 500 recalled lists from the same number of subjects in the age range of 35 to 55 years with an average of 45 years. These data are from Sample 1 when tested for the first time in 1988-1990. 
